# Transcriptomic inspection revealed a possible pathway regulating the formation of the high-quality brush hair in Chinese Haimen goat (*Capra hircus*)

**DOI:** 10.1098/rsos.170907

**Published:** 2018-01-10

**Authors:** Dejun Ji, Bo Yang, Yongjun Li, Miaoying Cai, Wei Zhang, Guohu Cheng, Haiyan Guo

**Affiliations:** College of Animal Science and Technology, Key Laboratory for Animal Genetics and Breeding of Jiangsu Province, Yangzhou University, Yangzhou, Jiangsu 225009, People's Republic of China

**Keywords:** goat, brush hair, differentially expressed genes, heat stress

## Abstract

The high-quality brush hair, or Type III brush hair, is coarse hair but with a tip and little medulla, which uniquely grows in the cervical carina of Chinese Haimen goat (*Capra hircus*). To unveil the mechanism of the formation of Type III brush hair in Haimen goats, transcriptomic RNAseq technology was used for screening of differentially expressed genes (DEGs) in the skin samples of the Type III and the non-Type III hair goats, and these DEGs were analysed by KEGG pathway analysis. The results showed that a total of 295 DEGs were obtained, mainly from three main functional types: cellular component, molecular function and biological process. These DEGs were mainly enriched in three KEGG pathways, such as protein processing in endoplasmic reticulum, MAPK, and complement and coagulation cascades. These DEGs gave hints to a possible mechanism, under which heat stress possibly initiated the formation. The study provided some useful biological information, which could give a new view about the roles of certain factors in hair growth and give hints on the mechanism of the formation of the Type III brush hair in Chinese Haimen goat.

## 1. Introduction

High-quality (Type III) brush hair is coarse hair that grows on the cervical carina of Haimen goats. Haimen goats, also named Yangtze River Delta white goats, are a unique indigenous Chinese goat breed raised for their specialized hair, which is used for making high-quality writing brushes. Brush hair is usually graded into three levels, primary (Type I), moderate (Type II) and high quality (Type III), based on the fibre diameter, length, colour, lustre and fine tip. Type I and II are normal coarse hairs with a medulla almost throughout the whole hair fibre, while Type III contains little medulla but a structurally fine tip. Little information is known regarding the genetic mechanism forming this unique hair type. Many studies have identified certain genes associated with the growth and properties of wool fibres in sheep and goats, such as *DSG1* [1], *IGF-IR* [2], *KRTAPs* [3], *ILK* [4], as well as the *KRT* and *KRTAP* genes [5]. In addition, androgens have been shown to upregulate the expression of insulin-like growth factor-I [6], while heat shock cognate protein, Hsc70 may be involved in androgen action on dermal papilla (DP) cells [7]. Research works on differential display at the mRNA [8] and proteomic levels [9] have revealed that the formation of Type III brush hair is initiated by androgens and heat stress, or in some cases cold stress, which activates and modulates protein synthesis involved in the formation stage of hair growth. In addition, RNA sequencing showed a difference in transcription at the cervical carina region in Type III goats and non-Type III goats where certain differentially expressed genes (DEGs) and functional pathways were analysed [10]. In the present report, through studying DEGs from RNA sequencing assays and several established signalling pathways, a possible mechanism for the formation of Type III brush hair is proposed.

## 2. Material and methods

### 2.1. Animals and sampling

**Figure 1.**
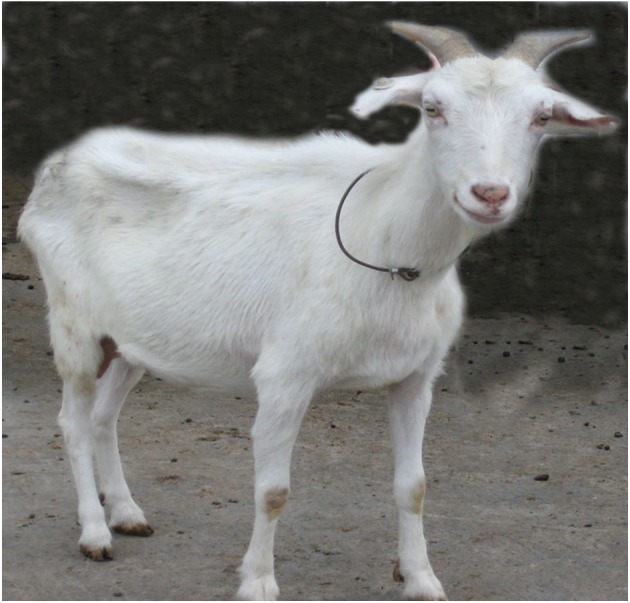
A Haimen goat ram.

Six half-sibling healthy male Yangtze River Delta white goats approximately 6 months old were bred under the same conditions and obtained from the state-operated Haimen Breeding Goat Farm, China (figure 1). The goats used in this study had an average body weight of 11.5 kg and hair diameter of approximately 35 µM. Skin tissue samples were collected from the cervical carina region of brush hair-producing goats. Skin samples from Group A were taken from the region where Type III hair grows, and samples were collected from three non-Type III goats where only Type I or Type II hair grows (designated as Group B). Hair was removed after applying local anaesthesia. A skin tissue sample of approximately 2 cm^2^ from the cervical carina region was excised using a scalpel. The skin tissue samples were then stored in liquid nitrogen (−196°C) for RNA sequencing.

### 2.2. RNA extraction, library preparation and sequencing

Total RNA was isolated using the Trizol Reagent (Invitrogen Life Technologies, USA), after which the concentration, quality and integrity were determined using a NanoDrop spectrophotometer (Thermo Scientific, USA). Three micrograms of RNA were used as input material for the RNA sample preparations. Sequencing libraries were generated using the TruSeq RNA Sample Preparation Kit (Illumina, San Diego, CA, USA). Briefly, mRNA was purified from total RNA using poly-T oligo-attached magnetic beads. Fragmentation was carried out using divalent cations under elevated temperatures in an Illumina proprietary fragmentation buffer. First-strand cDNA was synthesized using random oligonucleotides and SuperScript II. Second-strand cDNA synthesis was subsequently performed using DNA Polymerase I and RNase H. Remaining overhangs were converted into blunt ends via exonuclease/polymerase activities and the enzymes were removed. After adenylation of the 3′ ends of the DNA fragments, Illumina PE adapter oligonucleotides were ligated to prepare for hybridization. To select cDNA fragments of the preferred 200 bp in length, the library fragments were purified using the AMPure XP system (Beckman Coulter, Beverly, CA, USA). DNA fragments with ligated adaptor molecules on both ends were selectively enriched using an Illumina PCR Primer Cocktail in a 15-cycle PCR reaction. Products were purified using the AMPure XP system and quantified using the Agilent high-sensitivity DNA assay on a Bioanalyzer 2100 system (Agilent, USA). The sequencing library was then sequenced on a HiSeq platform (Illumina, USA) by Shanghai Personal Biotech. Co. Ltd, China.

### 2.3. Acquisition of the raw data, unigene cluster and annotation analysis

The original sequence data, or raw data reads, were saved as a FASTQ file, which included detailed read sequences and quality information. FastQC was used for quality control analysis and to filter out ‘dirty’ raw reads, such as reads with adapters, reads with more than 10% unknown bases and low-quality reads, which were defined as reads having more than 50% bases with a quality value ≤ 5. The clean reads obtained were used for subsequent analyses. De novo transcriptome assembly was performed using Trinity (Broad Institute of MIT and Harvard, USA). A K-mer library was constructed with the filtered reads, and the contigs were formed using Inchworm (Broad Institute of MIT and Harvard, USA). Using Chrysalis (Broad Institute of MIT and Harvard, USA), a component was built with the contigs, and de Bruijn graphs were generated. Then, Butterfly (Broad Institute of MIT and Harvard, USA) was used to optimize the de Bruijn graphs and create the final transcript through paths.

DEGs were then identified using a false discovery rate (FDR) ≤ 0.001 and an absolute value of log_2_Ratio ≥ 1 (two-fold change) as the significance thresholds. When the fold change is more than 2, and *p* is less than 0.05, it is designated as significant upregulation; when the fold change is less than 2 and *p* is less than 0.05, it is designated as significant downregulation. More stringent criteria, with a smaller FDR or greater fold-change value, were used for subsequent analyses. The DEGs were then compared to the sequences in the NR Protein, SwissProt database using BLAST and unigene annotations using the Blast2GO software (https://www.blast2go.com/).

### 2.4. Real-time quantitative PCR verification of differentially expressed genes

Six DEGs, *AOC3*, *DUSP1*, *IGFBP*, *VCL*, *WNK1* and *S100A7*, were selected, based on the DEG results and the change in expression between both groups, with *GADPH* as the reference, for verifying the transcriptomic results. RNA was reverse transcribed to cDNA using a cDNA Synthesis Kit (TIANGEN, Beijing, China). Primers are listed in table 1. Real-time qPCR was performed on an ABI7500 system (Applied Biosystem, CA, USA) using a qPCR Detection kit (SYBR Green) (TIANGEN, Beijing, China), within a 20 µl system : 10 µl 2 × SuperReal PreMix Plus, 0.6 µl each primer (10 µM); 1 µl cDNA template, 0.4 µl 50 × ROX Reference Dye, 7.4 µl RNase-Free ddH_2_O. Pre-denaturation was carried out for 15 min at 95°C, followed by 40 cycles of 10 s denaturation at 95°C, and finally, 32 s of annealing at 60°C. Data were processed using ABI7500 Software v. 2.0.6 (Applied Biosystem, CA, USA) and the relative expression amount was computed by the 2-Δ*Δ*Ct method. The qPCR detection was repeated three times, and was statistically analysed using *t*-test analysis (table 1) with standard deviation values. *p* < 0.05 was considered statistically significant.

**Table 1.** Primer pairs for real-time qPCR.

gene	access number	primer sequence (5′–3′)	products size/(bp)	annealing temperature/(°C)
*AOC3*	XM_005693871.1	F:ATCCAGACGCTGGCTGTGAC	196	60
		R:TGTTGGGAATGTCCTCTGCAT		
*DUSP1*	XM_005694581.1	F:TTTTCTGCTTCCTACCCGGAG	140	60
		R:GGGCCACCCTGATCGTAGAG		
*VCL*	XM_005699224.1	F:ATTCCCTGAGCAGAAAGCCG	108	59
		R:ATGATGTCATTGCCCTTGCTG		
*WNK1*	XM_005681041.1	F:AGAGATGCGTTTGTGGAGCA	153	61
		R:TTCCAATTTTTGGGCGCCTG		
*IGFBP1*	NM_001285763.1	F:GCGATGAGGCCACAGATACA	121	60
		R:TCCTCACTGGACTCGGTCAT		
*S100A7*	XM_005677509.1	F:CTAAGCTGGAGCAGGCCATT	142	60
		R:CCCCTTTTCTCACAGGCACT		
GAPDH*	XM_005680968. 1	F:GCAAGTTCCACGGCACAG	249	59
		R:GGTTCACGCCCATCACAA		

*GAPDH is used as control.

### 2.5. GO functional enrichment and pathway analysis

The Blast2go program was used to annotate unigenes on the basis of GO terms and NR database annotation. Conservation of gene identities of other species was analysed using BLASTX. To annotate genes with common denominators or functional categories, unigenes were also aligned with the eggNOG (evolutionary genealogy of genes: Non-supervised Orthologous Groups) database (http://www.ncbi.nlm.nih.gov/COG/, http://eggnog.embl.de/version_3.0). To summarize pathway information, the KEGG (Kyoto Encyclopedia of Genes and Genomes) Automatic Annotation Server (KAAS) was used to perform pathway annotation. Pathways with a Qvalue ≤ 0.05 were considered significantly enriched.

## 3. Results

### 3.1. Information of the transcriptomic sequencing

A total of 485 494 contigs were obtained with a mean length of 227 bp. Based on these contigs, 145 400 transcripts with a mean length of 539 bp were deduced, and annotation of these transcripts led to 32 812 unigenes with a mean length of 879 bp (GenBank accession number: SRP061582).

### 3.2. Differentially expressed genes between two groups

By comparison of the data between two groups, 295 DEGs were obtained: 132 upregulated and 62 downregulated. There were 101 DEGs expressed only in Type III group (Dryad, http://dx.doi.org/10.5061/dryad.12f01 [11]). The first 10 DEGs ranked according to the standard of two-fold change are listed in table 2. Other DEGs associated with hair growth and regulation included CaM (calmodulin), TUBA (tubulin alpha), FLNA (filamin alpha) UFD1 (ubiquitin fusion degradation protein 1), HSPs (heat shock proteins), SERPINC1 (the gene encoding antithrombin) and MARCH6 (E3 ubiquitin-protein ligase membrane-associated RING finger 6).

**Table 2.** DEGs with top 10 highest significant fold changes between two groups.

	unigene	Type III	non-Type III	fold change (−log_2_)
1	leucocyte cell-derived chemotaxin 2	42.0333	0.0746	8.64
2	apolipoprotein A-I	188.0913	0.4143	8.32
3	fibrinogen gamma chain	22.4539	0.0654	7.92
4	Hsp70	18.4728	0.0610	7.74
5	vinculin isoform 4	4.6669	0.0157	7.71
6	Mekk1	8.7731	0.0345	7.49
7	fibrinogen beta chain precursor	38.5539	0.1753	7.28
8	dual specificity protein phosphatase 6	29.8185	0.1650	6.99
9	PAB-dependent poly(A)-specific ribonuclease subunit 3	12.5222	0.0743	6.89
10	warm-temperature-acclimation-related 65-kDa protein	5.9871	0.0358	6.89

Note: an FDR ≤ 0.001 (FDR no greater than 0.05) and an absolute value of log_2_Ratio ≥ 1 (two-fold change) were regarded as the significance thresholds for DEGs.

### 3.3. GO functional enrichment

GO analysis of the 295 DEGs grouped genes into three main types: cellular component, molecular function and biological process, with a total of 104 subtypes. Eleven out of these were significant gathering subtypes (*p* < 0.05) (table 3).

**Table 3.** GO functional significant enrichment for different expressed genes.

main type	subtype	match (%)	*p*-value
cellular component	extracellular region	29 (9.83%)	0.012928
	cytoplasm	147 (49.83%)	0.047668
molecular function	structural molecule activity	48 (16.27%)	1.39 × 10^−18^
	RNA binding	28 (9.49%)	0.002263
biological process	translation	37 (12.54%)	5.14 × 10^−13^
	biosynthetic process	98 (33.22%)	0.013509
	ribosome biogenesis	7 (2.37%)	0.016254
	mRNA processing	13 (4.40%)	0.02431
	helicase activity	9 (3.05%)	0.026046
	peptidase activity	22 (7.46%)	0.026575
	generation of precursor metabolites and energy	9 (3.05%)	0.04366

### 3.4. KEGG pathway analysis

**Figure 2.**
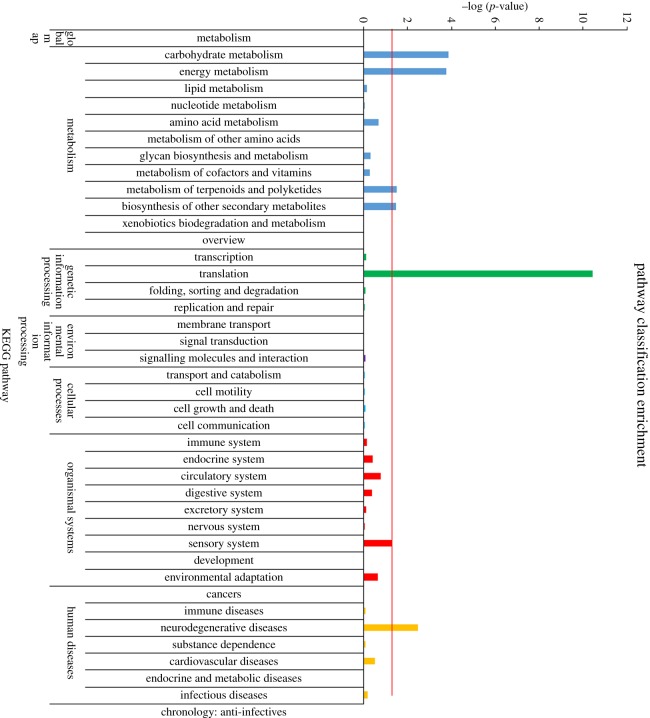
Pathway classification enrichment of the 295 DEGS.

Pathway classification enrichment analysis based on the KEGG database indicated that these DEGs were mainly enriched in four KEGG pathways, particularly in the translation pathway (figure 2). The main signalling pathways involving these DEGs were the following three pathways: protein processing in the endoplasmic reticulum, the MAPK signalling pathway and the complement and coagulation cascade (table 4).

**Table 4.** Main KEGG pathways for differentially expressed genes.

KEGG pathways	gene number
protein processing in endoplasmic reticulum	5
MAPK signalling pathway	4
complement and coagulation cascades	6

### 3.5. Quantitative PCR verification of the transcriptomic data

Quantitative PCR was conducted to verify the reliability of the transcriptomic results. Six genes, including four upregulated genes, *VCL*, *WNK1*, *DUSP1* and *AOC3*, and two downregulated genes, *IGFBP* and *S100A7*, with *GADPH* as the reference, were selected to test the transcript amount in samples from both groups.

**Figure 3.**
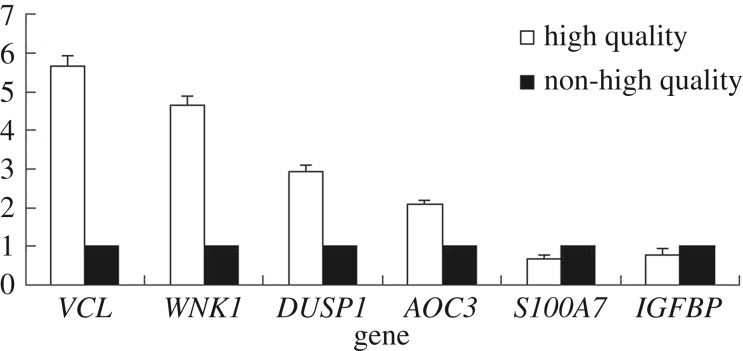
The relative expression of six genes. *VCL*, *WNK1*, *DUSP1* and *AOC3* were upregulated genes; *IGFBP* and *S100A7* were downregulated genes.

Using the transcript amount of the six genes in the non-Type III group as the reference value of 1, the relative transcript amount of the six genes with the value of standard deviation in the Type III group was 5.67 ± 0.25, 4.65 ± 0.25, 2.92 ± 0.18, 2.07 ± 0.11, 0.78 ± 0.16 and 0.66 ± 0.13, respectively. The relative differences for each gene between the two groups were identical with that deduced from the transcriptomic data (figure 3).

## 4. Discussion

### 4.1. Factors involved in the forming of the Type III brush hair

Type III brush hair is used for making first-class writing brushes due to its white colour, straight hair shaft, long and fine tip, bright lustre and good flexibility. It is a traditional export commodity popular in several nations in South and East Asia under the influence of Chinese culture, such as Japan, Korea and Singapore. Type III brush hair is only produced by Haimen goats, now a unique national resource breed used in producing Type III brush hair in China. Type III brush hair has been previously studied for its structural characteristics [12]. Particularly, studies have focused on growth regions of the animals [13] with an emphasis on the influence of environmental conditions and nutrition. It has been shown that a growth phase during puberty is critical to the formation of the Type III brush hair [12,13]. Specifically, androgens expressed at puberty activate protein synthesis commencing hair growth. Androgens also regulate sebaceous gland and hair growth by acting upon two different types of target cells, the epithelial sebocytes of sebaceous glands and the mesenchymal cells of the hair follicle DP [14]. In addition, without elevated androgen levels, initiation of Type III brush hair growth might be stunted [13].

### 4.2. Related genes from previous studies

Various growth phases of hair result in differential gene expression. Related research on wool and cashmere showed that Fibroblast Growth Factor 5 (FGF5) acted as an inhibitor in the regulation of the anagen–catagen phase transition of cashmere goat DP cells [15]. In addition, other genes, such as *STC2*, *VEGFR* and *ROR2* functioned in the process of cell differentiation and in the cell cycle [16]. Previous studies conducted on Type III brush hair revealed that seven genes were involved in the formation of Type III brush hair [8]. This included four previously characterized genes: CKLF-like MARVEL transmembrane domain-containing protein 3 (*CMTM3*), S100 calcium-binding protein A (*S100A*), protein kinase inhibitor gamma (*PKIG*) and fibulin 1-D. These data agree with the transcriptomic results presented in this study. *S100A* may play an important role in the activation of stem cells at the onset of hair follicle regeneration [17]. The *PKIG* gene may participate in the regulation of hair growth by inhibiting the activity of protein kinase A, which may regulate hair follicle activity *in vivo* [18]. Fibulin 1-D protein is putatively associated with basement membranes and elastic extracellular matrix fibres [19]. The results in this study were identical with the established proteomic results [9], but with much higher resolution.

### 4.3. Regulatory pathway on formation of the Type III brush hair

Previous research has established that Type III brush hair only grows during 6–8 months of age, or at the puberty stage, in Haimen goats. This implies a role of androgens in the hair growth process. Additionally, activation of androgens, and the androgen receptor, requires expression of HSPs. However, the expressions of androgen genes in Type III and non-Type III groups were not statistically different. These results demonstrated that high levels of androgen are important for hair growth, but need the coordination of other factors for the specific production of the Type III brush hair.

**Figure 4.**
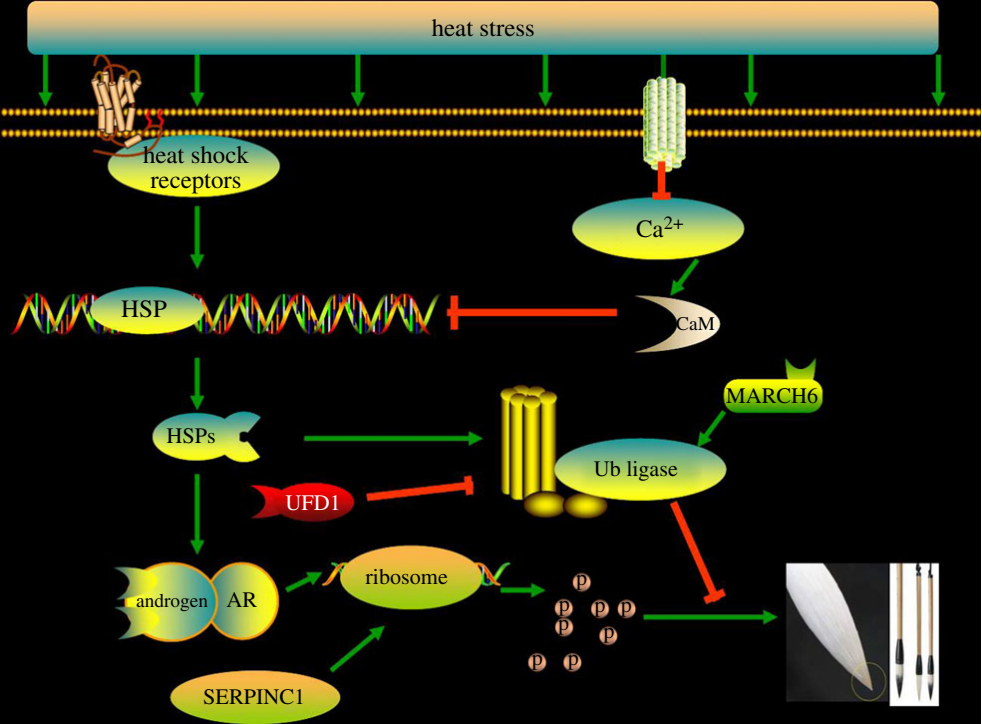
A possible pathway that regulates the formation of the Type III brush hair (partly from [20]). The linking arrows stand for positive regulation, while the line with plain ends mean negative regulation.

The difference in heat stress-associated genes between the two groups, however, may provide insight into the pathway of Type III hair growth. Heat stress could affect intracellular free calcium ion (Ca^2+^) levels and hence the accumulation of calmodulin. The lower expression of calmodulin and higher expression of HSPs in the Type III group in this study indicated that heat stress could be the key initiator in the formation of the Type III brush hair under a relatively higher level of androgens. Previous observations showed behavioural depression in some goats that produced the Type III brush hair. One possibility is that cold stimulus, rather than heat, initiates Type III brush hair. This is postulated based on the lower expression of calmodulin and higher expression of HSPs. Higher temperatures could induce higher levels of both CaM and HSPs (figure 4). Calmodulin is an important activator of Hsp70 in a dose-dependent manner in order to compete against HSF1-Hsp70 in forming the CaM-Hsp70 complex. The activation of Hsp70 could promote binding to novel peptides still present in the ribosome through the C- terminal end, preventing premature folding. Another possibility is participation in the regulation of the ubiquitin ligase complex mediated by MARCH6 [21]. This may occur in the early phase of Type III brush hair formation by effecting the rapid polymerization of tubulin, filamin and other monomer hair components. This would require the help of anticoagulation factors (SERPINC1). This postulated process has been verified in a preliminary manner due to the significantly lower expression of CaM, TUBA, FLNA and UFD1 (ubiquitin fusion degradation protein 1), and higher expression of heat stress-associated genes (*Hsp70*, *Hsp90* and *Hsp110*), SERPINC1 (F2, or antithrombin), FGB/G (fibrinogen beta/gamma chain precursor), MARCH6 and other signals. The final effect of this regulation would lead to the formation of hair with little medulla in goats (figure 3).

The deduced pathway given in this paper is still under investigation. The proposed pathway does not provide data for how MARCH6 is upregulated. In addition, further analysis of DEGs between the Type III and the non-Type III group is needed. Although the differential expression analyses indicated a possible mechanism in the formation of the Type III brush hair, direct evidence of HSP regulation of hair polymerization is lacking. Specifically, the pathway from higher levels of protein synthesis and ubiquitination to the formation of the Type III brush hair is still lacking molecular evidence for the assembly process of hair components. Thus, further work in this field is still needed.

## 5. Conclusion

This study provided the transcriptomic properties and DEGs in Type III brush hair-producing and non-Type III brush hair-producing goats. A further GO functional analysis was given along with KEGG pathway analysis. In this manner, a possible pathway was deduced to form the valuable Type III brush hair based on the transcriptomic data. These findings will be helpful in order to use goats capable of producing the valuable Type III brush hair.

## Data accessibility

Our data are deposited at GenBank (accession number SRP061582) and Dryad (http://dx.doi.org/10.5061/dryad.12f01) [11].
